# In vivo effects of olive oil and trans-fatty acids on miR-134, miR-132, miR-124-1, miR-9-3 and mTORC1 gene expression in a DMBA-treated mouse model

**DOI:** 10.1371/journal.pone.0246022

**Published:** 2021-02-04

**Authors:** Richard Molnar, Laszlo Szabo, Andras Tomesz, Arpad Deutsch, Richard Darago, Nowrasteh Ghodratollah, Timea Varjas, Balazs Nemeth, Ferenc Budan, Istvan Kiss

**Affiliations:** 1 Department of Public Health Medicine, Medical School, University of Pécs, Pécs, Hungary; 2 Institute of Physiology, Medical School, University of Pécs, Pécs, Hungary; 3 Institute of Environmental Engineering, Faculty of Engineering, University of Pannonia, Veszprém, Hungary; 4 Szentagothai Research Centre, University of Pécs, Pécs, Hungary; Semmelweis University, HUNGARY

## Abstract

Both the intake of beneficial olive oil and of harmful trans-fatty acids (TFAs) in consumed foods are of great significance in tumor biology. In our present study we examined the effects they exert on the expression patterns of miR-134, miR-132, miR-124-1, miR-9-3 and mTOR in the liver, spleen and kidney of mice treated with 7,12-dimethylbenz [a] anthracene (DMBA). Feeding of TFA-containing diet significantly increased the expression of all studied miRs and mTORC1 in all organs examined, except the expression of mTORC1 in the spleen and kidney. Diet containing olive oil significantly reduced the expression of miR-124-1, miR-9-3 and mTORC1 in the liver and spleen. In the kidney, apart from the mTORC1 gene, the expression of all miRs examined significantly decreased compared to the DMBA control. According to our results, the cell membrane protective, antioxidant, and anti-inflammatory effects of olive oil and the cell membrane damaging, inflammatory, and carcinogenic properties of TFA suggest negative feedback regulatory mechanisms. In contrast to our expectations, mTORC1 gene expression in the kidney has not been shown to be an appropriate biomarker–presumably, because the many complex effects that regulate mTOR expression may quench each other.

## Introduction

Malignant tumorous diseases are the second leading causes of deaths worldwide; according to the estimates of WHO they caused 9.6 million deaths in 2018. The main causes of the development of these diseases are the adverse environmental effects [1], within which eating habits represent a major factor [2]. Such a factor, for example, is the intake of fatty acids (FAs) including harmful trans-fatty acids (TFAs) [3, 4]. On the other hand, olive oil, which has beneficial effects and is rich in antioxidant and antitumor oleic acid and contains approximately 10% linoleic acid, is also widely consumed [5, 6].

The amount of daily TFA intake shows positive correlation with mortality when comparing the data of the upper (≥ 2.73 calorie percent) and lower quartiles (≤1.41 caloric percent) on the basis of the hazard ratio (HR) normalized to gender and age (HR: 1.03; CI 1.00–1.05; p trend = 0.0062) [4]. Although TFA intake does not correlate with overall cancer mortality, there is a positive correlation between the regular daily intake of TFA and relative risk (RR) of breast cancer in postmenopausal women (RR: 1.37; 95% CI 1.04–1.81; p = 0.02) [4, 7]. The adverse effects of TFA are also shown by the fact that a 2% increase in dietary caloric intake significantly increased the risk of cardiovascular diseases (RR 1.23; 95% CI 1.11–1.37; p <0.001) [8].

Indeed, there are direct and indirect harmful molecular mechanisms associated with TFA consumption. For example, trans-linoleic acid (trans, trans-9-12-octadecadienoic acid) (TLA) and elaidic acid (trans-9-octadecenoic acid) (EA), which belong to TFAs, increase the amount of intercellular adhesion molecule-1 (ICAM-1) and vascular cell adhesion molecule-1 (VCAM-1), among other [9]. Both ICAM-1 and VCAM-1 generate reactive oxygen species (ROS), which activate nuclear factor kappa B (NF-κB)–which has direct pro-inflammatory effect [9]. Its further significance is, that in addition to inflammatory signaling pathways, NF-κB activation may be associated with malignant transformation processes, as well [10].

When studying the protective effects of olive oil Pelucchi et al. have performed a meta-analysis on the relationship between olive oil and cancer, where the calculated summary relative risk of breast cancer was 0.62 (95% CI 0.44–0.88) for the highest versus the lowest level of olive oil consumption [3]. Furthermore, in a case-control study, olive oil consumption significantly reduced the risk of development of lung cancer (OR: 0.65; 95% CI: 0.42–0.99; p <0.05) [11]. In another case-control study performed by Bosetti et al., a significant trend in the protective effect against laryngeal cancer was observed between the upper quartile consuming 42.9 grams of olive oil per day and the lower quartile consuming less than 3.2 grams per day (OR: 0.4; 95% CI: 0.3–0.7; p = 0.01) [2]. In another case-control study when comparing the lowest versus the middle tertile consuming less than 1.6 grams of olive oil per day (OR: 0.62; 95% CI: 0.39–0.99), and the highest versus the lowest tertile consuming above 3.9 grams per day (OR: 0.47; 95% CI: 0.28–0.78; p-trend = 0.002), a statistically significant inverse dose-response association was found between development of bladder cancer and the olive oil consumption [12].

MicroRNAs (miRNA) bind to the 3 ’UTR of mRNAs and thus reduce the translation of mRNAs–and thereby through gene silencing affect the protein synthesis, the cell cycle [13], apoptosis, or even cell differentiation [14]. MiRNAs may serve as early molecular epidemiological biomarkers for the detection of malignancies [15]. In addition, TFA also causes oxidative stress and lipid peroxidation [16], which may be associated with the expression pattern of certain miRNAs (e.g., miR-134), as miR-134 is involved in tumor cell proliferation, apoptosis, invasion, and also in the regulation of metastasis formation [17]. Furthermore, miR-134 is known as a tumor suppressor because it directly silences the KRAS oncogene as well as the integrin beta 1 (ITGB1) oncogene [18, 19], the activity of which genes also promotes malignant transformation and proliferation of malignant cells [20], which can lead to renal cell carcinoma (RCC) [21]. MiR-132 also inhibits proliferation in hepatocellular carcinoma (HCC) by inactivating the AKT / mTOR signaling pathway [22], and by inhibiting IL1β and IL6 expression through inhibition of the transcriptional co-activator P300 [23]. Overexpression of miR-124 was observed in HCC for the regulation of proliferation of the anti-apoptotic “baculoviral IAP repeat containing 3 (BIRC3) protein”, as well as for the inhibition of NF-κB signaling pathway and *C-MYC* oncogene expression [24]. (For general expressions of examined miRs and *mTOR* see Table 1, for the list of abbreviations Table 2).

**Table 1:** General expressions of examined miRs and *mTOR*.

	miR-134	miR-132	miR-124	miR-9	mTOR	Literature
**DMBA**	+	+	+	+	+	
**TFA**	+	+	+	+	+	
**Olive oil**	-	-	-	-	-	
**HCC**	-	-	- or +			[19, 24]
**RCC**	-			+	+	[19, 46]

**Table 2:** List of abbreviations.

Abbreviation	Name of expression
ATM	ataxia-telangiectasia mutated
BIRC3	baculoviral IAP repeat containing 3
DMBA	7,12-dimethylbenz [a] anthracene
EA	elaidic acid
FA	fatty acid
GSH	gluthation
HCC	hepatocellular carcinoma
HIF-1α	Hypoxia-inducible factor 1-alpha
HR	hazard ratio
ICAM-1	intercellular adhesion molecule-1
IL1β	interleukin 1β
IL6	interleukin 6
ITGB1	integrin beta 1
miRNA	microRNA
MMP	matrix metalloproteinase
mTOR	mammalian target of rapamycin
NF-κB	nuclear factor kappa B
PTEN	phosphatase and tensin homolog deleted on chromosome 10
PUFA	poly unsaturated fatty acids
RCC	renal cell carcinoma
ROS	reactive oxygen species
RR	relative risk
TFA	trans-fatty acid
TGF-β	transforming growth factor-β
TLA	trans-linoleic acid
TNF	tumor necrosis factor
TSC2	tuberous sclerosis complex 2
VCAM-1	vascular cell adhesion molecule-1

Thus, miR-9, miR-124, miR-132, and miR-134 exert their antitumor activity indirectly, through the inhibition of NF-κB and AKT/mTOR signaling pathways [19, 22, 24, 25]. The mammalian target of rapamycin (mTOR) signaling protein plays a vital role in cellular functions such as cell proliferation, cell growth, protein synthesis, and its expression is influenced by a number of factors (see above). Thus, the question arises whether in addition to the miRs studied, the expression of *mTOR* can be used as a biomarker of carcinogenic and chemopreventive effects, or not.

In our present study we modelled two types of human dietary habits in mice, namely, high olive oil consumption and high TFA intake. For this purpose, we used a model developed by Tomesz et al., and we examined the expression of miR-134, miR-132, miR-124-1, miR-9-3, and of *mTOR* in the liver, spleen and kidney of 7,12-dimethylbenz [a] anthracene (DMBA) treated mice as molecular epidemiological biomarkers [26]. The effect of DMBA damage is indicated by an increase both in the expression of these miRs and in the expression of mTOR, among other, in the examined organs, since DMBA is a pluripotent carcinogen, while induces mutations and increases the expression of oncogenes, etc. [26]. The aim of our research was to explore the expression patterns of these miRs so that we can follow the harmful and beneficial tumor biological effects of TFA and olive oil.

## Materials and methods

In the experiment we used 6 to 8 weeks old CBA/Ca female mice, each group caged separated. For 14 days one group of animals (n = 6) was fed with olive oil in a dose of 0.3 g/animal/day (Agraria Riva Del Garda SCA) and another group (n = 6) received TFA (trans-3-hexadecenoic acid) (Sigma Aldrich) in a daily dose of 0.3 g/animal, mixed into their diet. The animals were treated once with DMBA 20 mg/kg body weight intraperitoneally, solved in 0.1 ml corn oil (Sigma Aldrich). In addition, a positive control group (n = 6) was given DMBA alone, as mentioned. Twenty-four hours after DMBA exposure, the animals were sacrificed by cervical dislocation and then their liver, kidney, and spleen were removed. The experiment was conducted in compliance with the current ethical regulations and approved by Regional Animal Ethical Committee Pécs (Ethical license no.: BA02/2000-79/2017).

### Isolation of total RNA

Total cellular RNA was isolated using TRIZOL reagent (MRTR118-20 Nucleotest Bio Ltd.) according to the manufacturer’s instructions. The quality of RNA was checked by NanoDrop absorption photometry and only RNA fractions with A> 2.0 at 260/280 nm were used for the RT-PCR process.

#### Reverse Transcription-Polymerase Chain Reaction (RT-PCR)

The one-step PCR, including reverse transcription and target amplification, was performed using Kapa SYBR FAST One-step RTQCR kit (Kapa Biosystems) in a 96-well plate, on a LightCycler 480 qPCR platform, according to the manufacturer’s instructions.

Temperature program was set as follows: 5 minutes incubation at 42°C, followed by a 3 minute incubation at 95°C, then 45 cycles were performed (95°C– 5 s, 56°C– 15s, 72°C– 5s) and a fluorescent reading was made at the end of each cycle. Each run was performed with melting curve analysis (95°C– 5s, 65°C– 60s, 97°C ∞) to confirm the specificity of the amplification. The reaction mixture was the following: 10 μl KAPA SYBR FASTqPCR Master Mix, 0.4 μl KAPA RT Mix, 0.4 μl dUTP, 0.4 μl primers, 5 μl miRNA template supplemented with sterile double-distilled water to a total volume of 20 μl.

Primer sequences for the *mTORC1* gene, the examined mRNAs (miR-330, miR-29a, miR-9-1, miR-9-3) and the internal control gene (mouse U6) are shown in Table 3. Primers were synthetized by Integrated DNA Technologies (Bio-Sciences), sequences are from previous publications [27, 28].

**Table 3:** Primer sequences (5’-3’) of the *mTORC1* gene, of the examined miRNAs (miR-330, miR-29a, miR-9-1, miR-9-3) and of the internal control gene (mouse U6).

	FORWARD	REVERSE
**miR-330**	GACCCTTTGGCGATCTCTG	CTGTGCTTTGCTCGTTGGAT
**mir-29a**	CCCCTTAGAGGATGACTGATTTC	AACCGATTTCAGATGGTGCT
**miR-9-1**	CGGGGTTGGTTGTTATCTTT	TGGGGTTATTTTTACTTTCGGTTA
**miR-9-3**	GCCCGTTTCTCTCTTTGGTT	TCTAGCTTTATGACGGCTCTGTGG
**mTORC1**	AAGGCCTGATGGGATTTGG	TGTCAAGTACACGGGGCAAG
**mouse U6**	CGCTTCGGCAGCACATATAC	TTCACGAATTTGCGTGTCAT

#### Calculation and statistical analysis

Relative miRNA expression levels were calculated and compared using the 2^-ΔΔCT^ method. During the statistical analysis for the testing the distribution of results we used the Kolmogorov-Smirnov test. To compare averages we used the Levene’s type F-probe and T-probe. IBM SPSS 21 statistical software was used for calculations and analysis. We determined the level of statistical significance at a p value <0.05.

## Results

**Fig 1:**
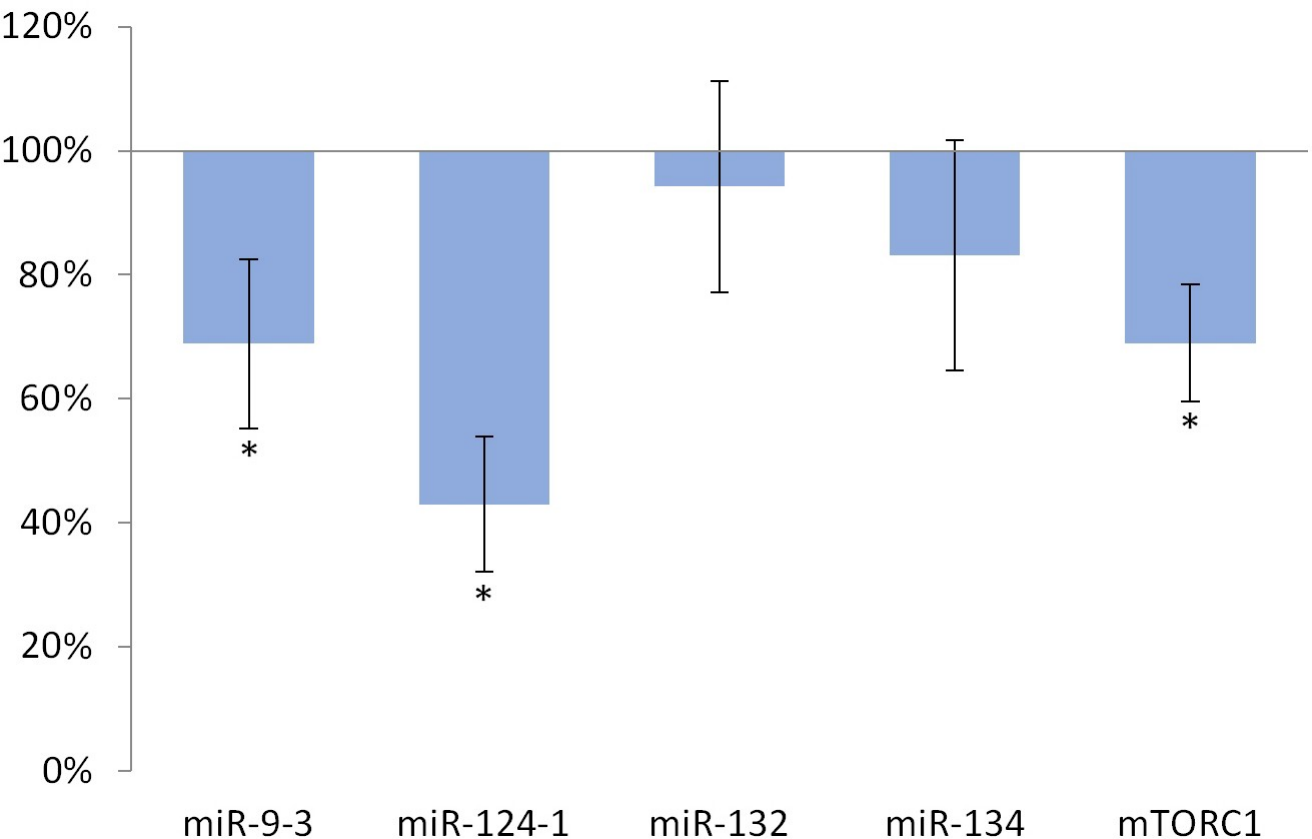
Liver of olive oil-consuming mice. The expression pattern of miR-134, miR-132, miR-124-1, miR-9-3, and *mTOR* relative to DMBA-induced expression in the liver of DMBA- and olive oil-treated mice.

Feeding with olive oil-containing diet significantly (p <0.001) reduced the expression of miR-124-1 (p = 0.001), miR-9-3 (p = 0.035), and *mTORC1* (p = 0.002) compared to the DMBA control (Fig 1).

**Fig 2:**
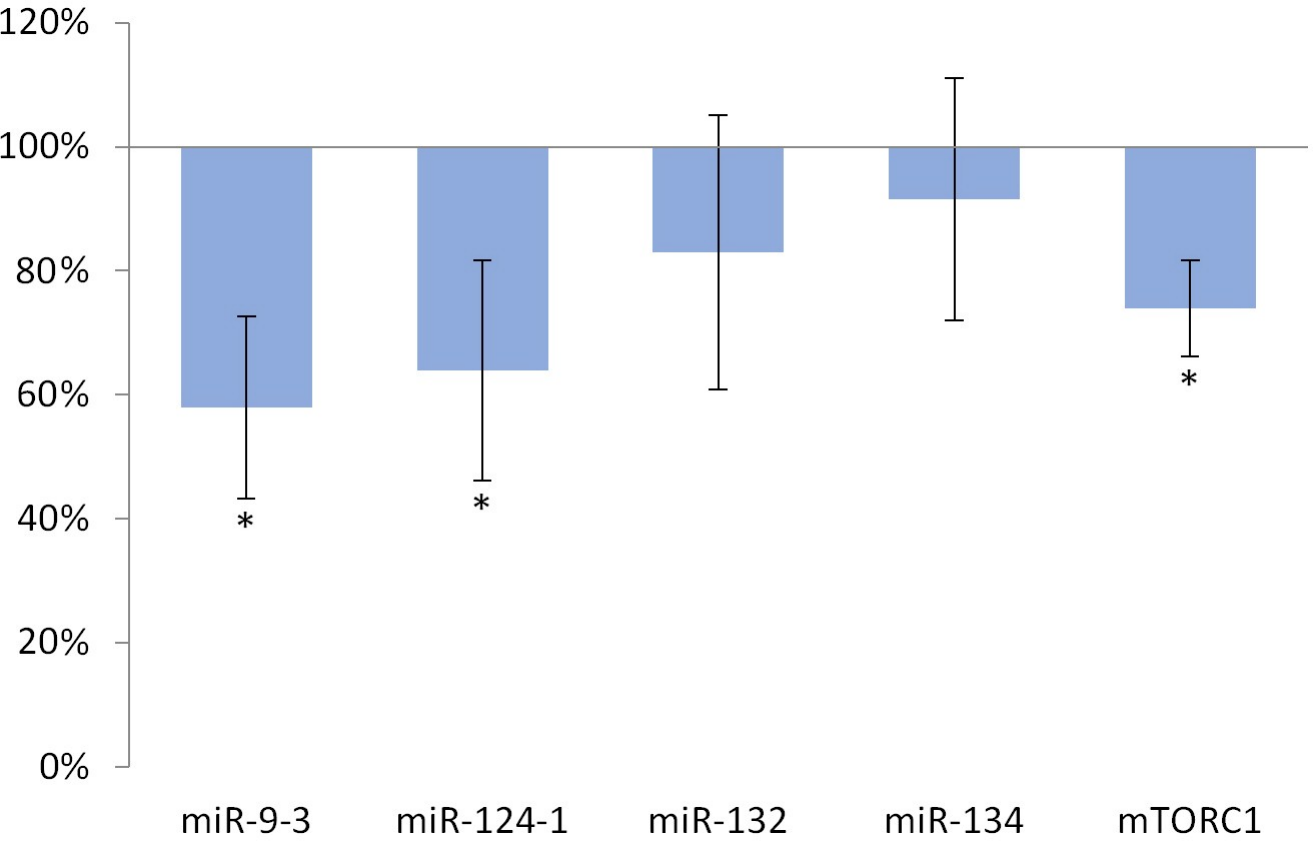
Spleen of olive oil-consuming mice. The expression pattern of miR-134, miR-132, miR-124-1, miR-9-3 and *mTOR* relative to DMBA-induced expression in the spleen of DMBA- and olive oil-treated mice.

Similarly, the expression of miR-124-1 (p = 0.034), miR-9-3 (p = 0.009) and *mTORC1* (p = 0.003) significantly decreased in the spleen as a result of the above mentioned feeding (Fig 2).

**Fig 3:**
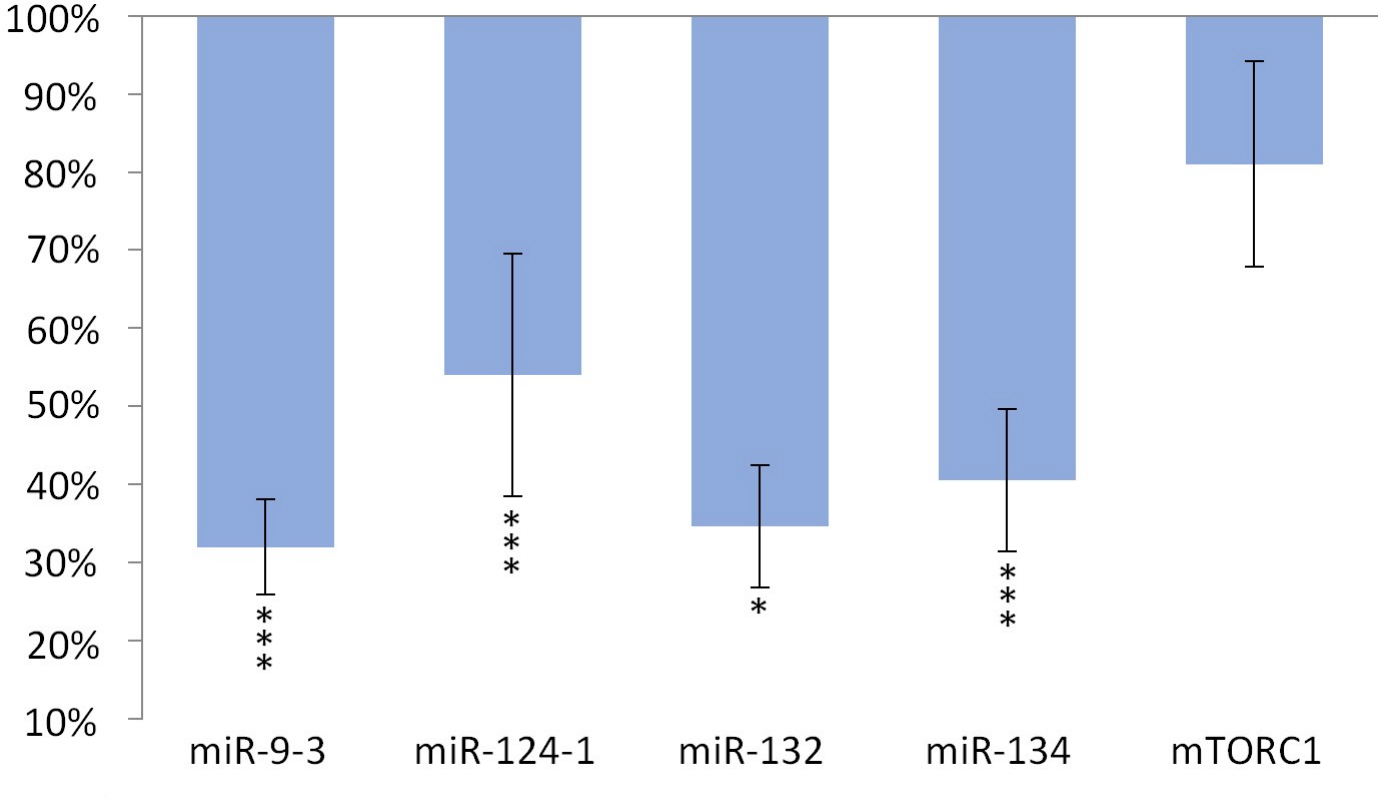
Kidneys of olive oil-consuming mice. The expression patterns of miR-134, miR-132, miR-124-1, miR-9-3, and *mTOR* relative to DMBA-induced expression in the kidneys of DMBA- and olive oil-treated mice.

The expression of miR-134 (p <0.001), miR-132 (p <0.001) and miR-9-3 (p <0.001) and miR-124-1 (p = 0.01), but not of *mTORC1* gene, significantly decreased in the kidneys compared to the DMBA control (Fig 3).

**Fig 4:**
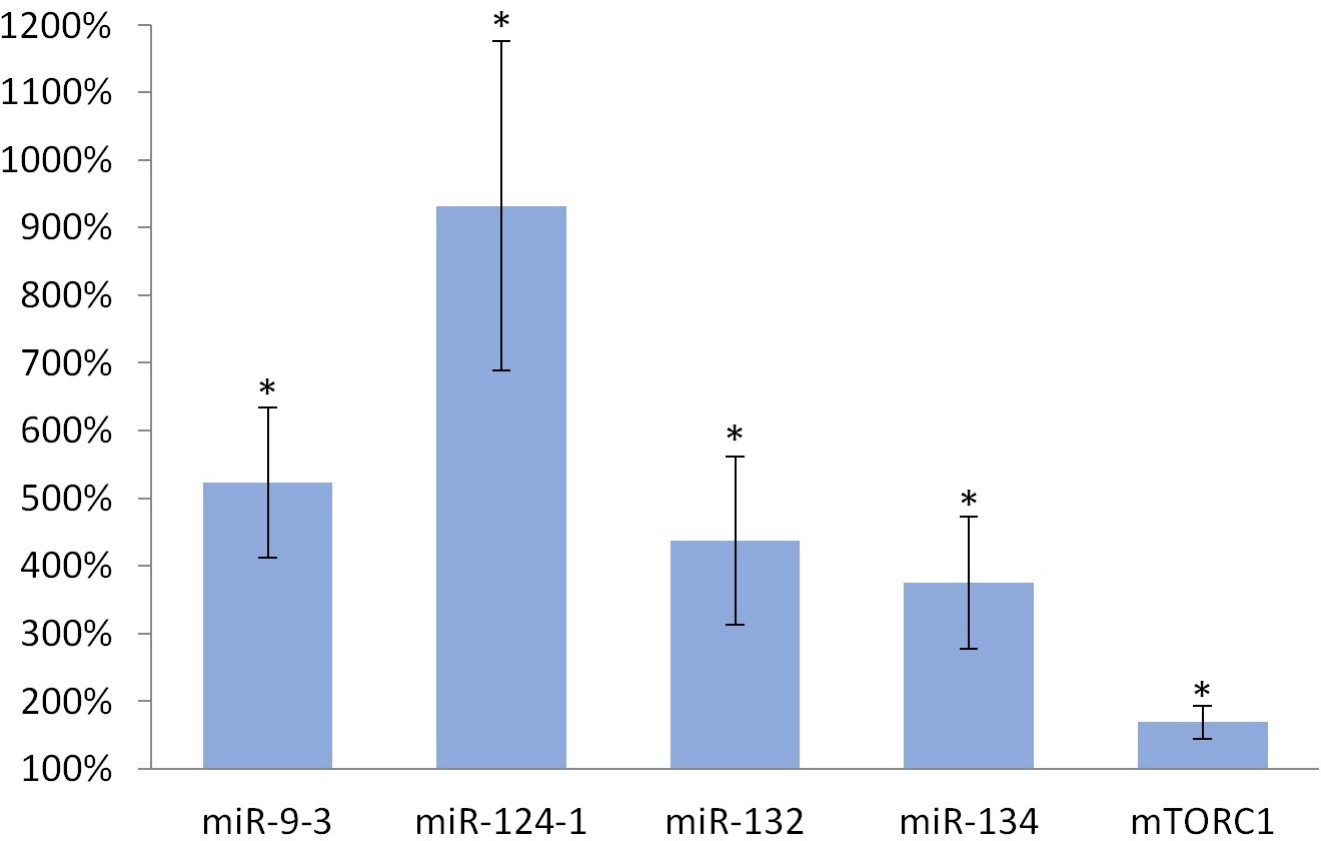
Liver of olive TFA-treated mice. The expression patterns of miR-134, miR-132, miR-124-1, miR-9-3 and *mTOR* relative to DMBA-induced expression in the liver of DMBA- and TFA-treated mice.

Consumption of TFA-containing diet significantly increased the expression of miR-134 (p <0.001), miR-132 (p <0.001), miR-124-1 (p <0.001), miR-9-3 (p <0.001) and *mTORC1* (p <0.001), as well, in the liver of animals compared to the DMBA control (Fig 4).

**Fig 5:**
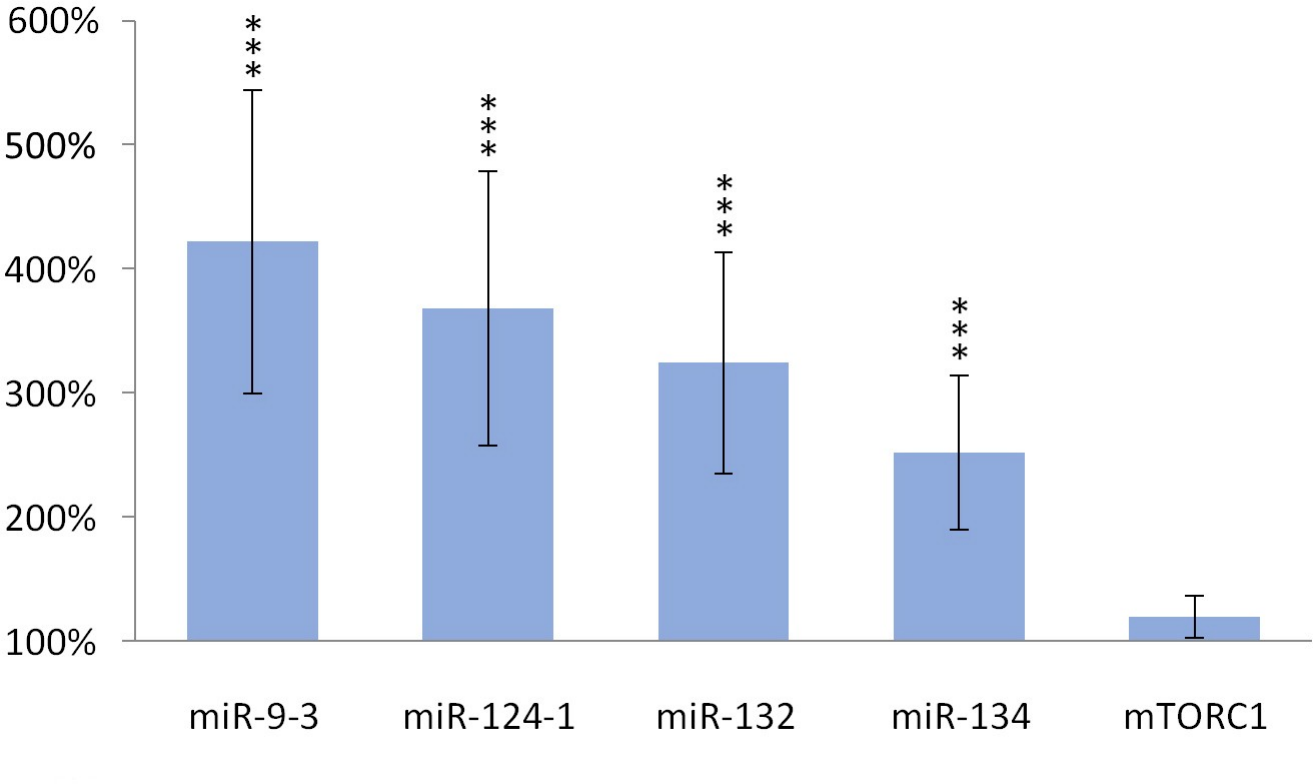
Spleen of olive TFA-treated mice. The expression patterns of miR-134, miR-132, miR-124-1, miR-9-3 and *mTOR* relative to DMBA-induced expression in the spleen of DMBA- and TFA-treated mice.

**Fig 6:**
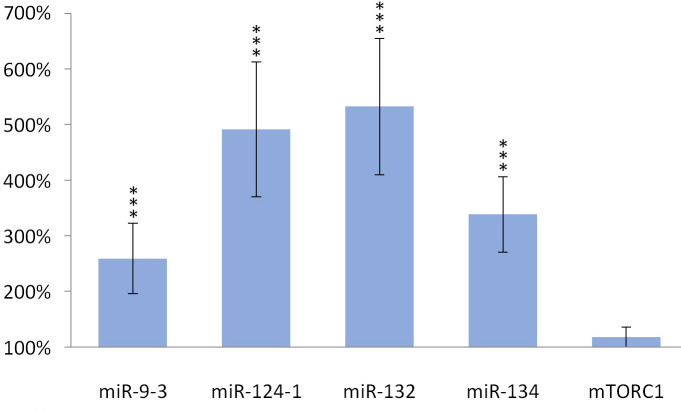
Kidneys of olive TFA-treated mice. The expression patterns of miR-134, miR-132, miR-124-1, miR-9-3 and *mTOR* relative to DMBA-induced expression in the kidneys of DMBA- and TFA-treated mice.

TFA also significantly (p> 0.001) increased the expression of miR-134 (p> 0.001), miR-132 (p> 0.001), miR-124-1 (p> 0.001), miR-9-3 (p> 0.001) in the spleen and kidneys compared to the DMBA control, but the gene expression of *mTORC1* was not significantly increased (Figs 5 and 6).

## Discussion

Oleuropein and oleocanthal, the water-soluble polyphenols of olive oil are absorbed from the small intestine and reach the spleen and liver [29], where they exert a protective effect against free radical-induced oxidative stress [30, 31] mainly through their cell membrane protective properties [32]. Oleuropein is able to inhibit NF-κB activation [33, 34] and to increase intracellular GSH levels, which is usually reduced by the free radicals [35–37]. This may lead to a decrease in the expression of miR-134, miR-132 and miR124-1 (via the mentioned negative feedback mechanisms) and to a significant reduction in the amount of overexpressed miR-9-3 associated with the DMBA treatment (Figs 1–3). This is supported by the direct β-catenin inhibitory effect of PUFA, that leads to a significant decrease of C-MYC [38]. Furthermore, P300 and miR-132 cross-regulate each other’s expression [23]. Expression of BIRC3 and miR-124 showed a negative correlation [24] suggesting a negative feedback loop, while ICAM-1 positively modulates miR-124 expression [39]. These data explain the significant decrease of miR-124 in the olive oil-consuming group in all organs examined, and its significant overexpression in the TFA consuming group (since both the earlier mentioned TLA and EA increase the amount of ICAM-1) [9] (Figs 1–6). Certainly, the protective effect of water-soluble oleuropein caused the strong and significant decrease of miR-134 and miR-132 in the kidney via the mentioned negative feedback regulation [39] (Fig 3). The significant decrease of *mTOR* gene expression seen both in the liver and in the spleen was also due to the potent inhibitory effect of oleocanthal on mTOR activity [40] (Figs 1 and 2).

The weaker, non-significant decrease in miR-134 and miR-132 and the significant decrease in miR-124 in the liver and spleen (Figs 1 and 2) in the group consuming olive oil seem to contradict the antitumor effect of olive oil, since reduced expression was also observed for miR-134, miR-132, and miR-124 in manifest HCC [19], as well as for miR-134 in RCC [19]. (The expression of miR-124 in HCC is contradictive Table 1, [19, 24]). In addition, miR-9 also inhibits HCC progression [25], and it has been shown that a decrease in miR-9-3 indicates the development of malignant tumors as an early biomarker [41]. However, DMBA-induced elevated expression of MYC [42] and MYCN oncoproteins cause an increase of miR-9 expression in tumor cells, which (this time via a positive feedback mechanism, supporting oncogenes)–through the amplification of E-cadherin–induces further increase of C-MYC expression [43, 44]. This, in contrast to the above, promotes the formation of HCC, that is supported by the result of our previous article [26], where miR-9-3 expression of female CBA/CA mice as a result of DMBA exposure resulted in a particularly large, significant increase. mTOR signaling pathway activating mutations have already been identified in a wide range of human malignancies [45], for example in RCC [46]. Activation of the PI3-K/Akt/mTOR signaling pathway induces a number of oncogenic processes that contribute to the growth, survival, and proliferation of tumor cells, for example cyclins, *C-MYC* and ornithine decarboxylase [46]. Also mTOR in RCC cells through phospholipase D enhances the expression of both Hypoxia-inducible factor 1-alpha (HIF-1α) and HIF-2α [46, 47]. On the other hand, DNA damage inhibits mTORC1 through activting p53-dependent transcription, as well as *TSC2* and *phosphatase and tensin homolog deleted on chromosome 10* (*PTEN)* [47]. Thus, our results with the olive oil consuming group are explained by the negative feedback mechanisms, that are often involved in the regulation of the expression of miRs [48] (Figs 1 and 3).

However, the metabolism of DMBA leads to the appearance of reactive oxygen species (ROS) [49, 50] which contributes to the harmful effects of TFAs [8]. Furthermore, ROS has also been shown to induce cytokines (TNF, IL1, IL6), to increase the amount of specific transcription factors (e.g., NF-κB) and to reduce the level of protective GSH [9, 51].

In addition, if IL1β is present in high amounts, it stimulates inflammatory growth factors such as TNF, matrix metalloproteinases (MMPs), etc. [52]. Both the MMPs and TNF (in a redundant manner) promote the malignant transformation of cells, as well as their progression [53], e.g., by activation of NF-κB [52, 54, 55], which inhibits the expression of the anticancer miR-134 and P53 genes [56]. Indeed, DMBA-treated mice showed increased levels of interleukin 1β (IL1β), interleukin 6 (IL6), and tumor necrosis factor (TNF), which ultimately increased the possibility of malignant transformation [57]. These effects may explain the effect of TFA in the organs examined (Figs 4–6).

**Fig 7:**
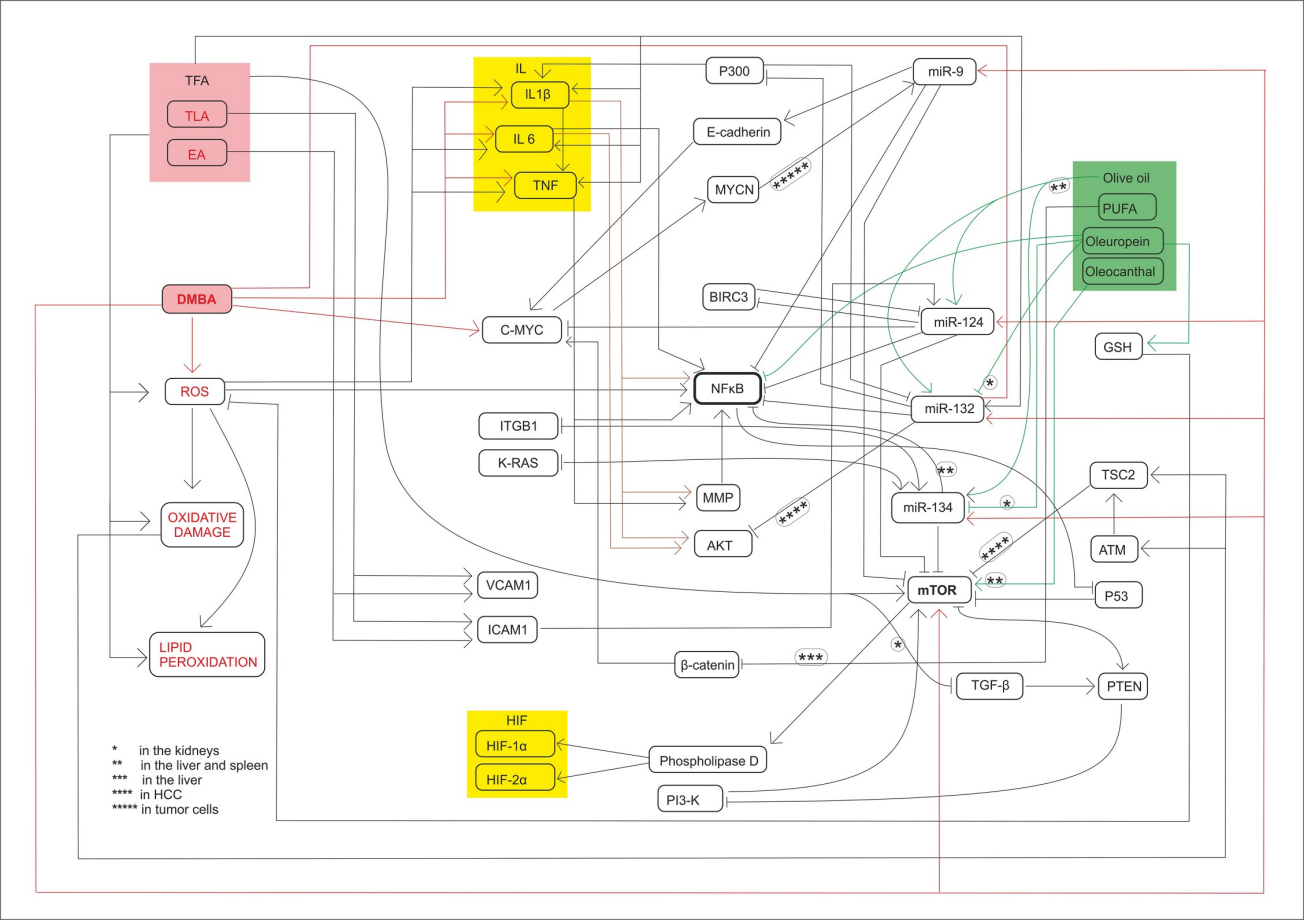
Summary of molecular mechanisms and signl transductions. The harmfull effects induced signal transductions and the chemopreventive effects too influence the expression of miR-134, miR-132, miR-124-1, miR-9-3 and *mTOR*.

It can generally be stated that in each of the organs examined in the group consuming TFA-containing diet, the miRs tested showed a nearly opposite expression pattern compared to the groups consuming olive oil. This is also consistent with the negative feedback mechanisms reported in the literature [26, 58], as well as with the probable negative feedback mechanisms [59, 60]. The only exception is the expression of the *mTOR* gene in the kidneys, which can be attributed to the resultant of multiple effects. On the one hand, as TFA may have induced *mTOR* expression in the liver of mice, it is also TFA that blocks transforming growth factor-β (TGF-β) receptors in the kidneys, leading to a decrease in PTEN [26]. PTEN through the inhibition of PI3-K is an inhibitor of MTORC1, as well–moreover, according to our present knowledge this inhibition lacks negative feedback [26]. Furthermore, tumor suppressor effect of “tuberous sclerosis complex 2” protein (TSC2) activated by ROS induced “ataxia-telangiectasia mutated” (ATM) protein may have also decreased the expression of *mTORC1* gene [61]. Our results in the spleen as well as in the kidneys of the studied animals consuming TFA show, that these multiple effects regulating the expression of the *mTORC1* gene balance each other–also taking into account the related mTOR-decreasing effect exerted by the above mentioned miR-132 [62] (Fig 7).

Thus, these effects causing constitutive transcriptional activation and proto-oncogene to oncogene mutations–and the corresponding miR expression pattern–are specific to manifest carcinomas (and to in vitro cancer cell cultures) [19, 21, 22, 24]. However, the miR and *mTOR* gene expression patterns in our study, as early biomarkers, can rather be considered as responses to biological effects on the organs studied.

## Conclusions

The expression of miR-134, miR-132, miR-124-1, and mir-9-3 indicated the chemopreventive effect of olive oil, as well as the carcinogenic effect of TFA (Figs 1–6). Our results confirmed the central role of inducible inflammatory signaling pathways among the mechanisms of the effects of different types of FAs on tumorigenesis. This is also the case for the expression pattern of both miRs and the mTOR gene, that is for example supported by ROS, NF-κB activated by inflammatory signaling agents, PTEN, and the level of accumulated ICAM-1 protein may have a key role [63]. We can conclude, that the expression patterns of the miR-134, miR-132, miR-124-1, mir-9-3, and *mTORC1* genes, as early biomarkers of carcinogenic and chemopreventive effects, differ from the expression patterns of manifest tumors and in vitro cell cultures Table 1, [19, 21, 22, 24, 45, 46]. In general, our results suggest the great importance of negative feedback regulatory mechanisms. This important observation draws attention to the fact that gene expressions measured in tumors may be completely different from the expressions of the same genes in the period before tumor development.

However, in contrast to our expectations, in the animal model used in the present study design, the expression of the *mTORC1* gene in the kidneys did not prove to be a suitable biomarker–to indicate the potential chemopreventive or carcinogenic/co-carcinogenic effects of either olive oil or TF consumption (Figs 3 and 6). It is very likely that this is because mTOR is driven by effects that also play an important role in inflammatory biology and in the cell cycle–and consequently complex (and even partially cross-regulatory) effects [22], which can also quench each other’s gene expression effects.

## Supporting information

S1 Table The raw data of DMBA induced miRs and *mTOR* expression, influenced by TFA and olive oil. (XLS)
